# A Perspective on Cortical Layering and Layer-Spanning Neuronal Elements

**DOI:** 10.3389/fnana.2018.00056

**Published:** 2018-07-17

**Authors:** Matthew E. Larkum, Lucy S. Petro, Robert N. S. Sachdev, Lars Muckli

**Affiliations:** ^1^Neurocure Center for Excellence, Charité Universitätsmedizin Berlin & Humboldt Universität, Berlin, Germany; ^2^Centre for Cognitive Neuroimaging, Institute of Neuroscience and Psychology, University of Glasgow, Glasgow, United Kingdom

**Keywords:** feedback, feedforward networks, top-down processing, calcium spikes, apical dendrite, ultra-highfield fMRI, layer fMRI

## Abstract

This review article addresses the function of the layers of the cerebral cortex. We develop the perspective that cortical layering needs to be understood in terms of its functional anatomy, i.e., the terminations of synaptic inputs on distinct cellular compartments and their effect on cortical activity. The cortex is a hierarchical structure in which feed forward and feedback pathways have a layer-specific termination pattern. We take the view that the influence of synaptic inputs arriving at different cortical layers can only be understood in terms of their complex interaction with cellular biophysics and the subsequent computation that occurs at the cellular level. We use high-resolution fMRI, which can resolve activity across layers, as a case study for implementing this approach by describing how cognitive events arising from the laminar distribution of inputs can be interpreted by taking into account the properties of neurons that span different layers. This perspective is based on recent advances in measuring subcellular activity in distinct feed-forward and feedback axons and in dendrites as they span across layers.

## Conceptual Shift

**Figure 1:**
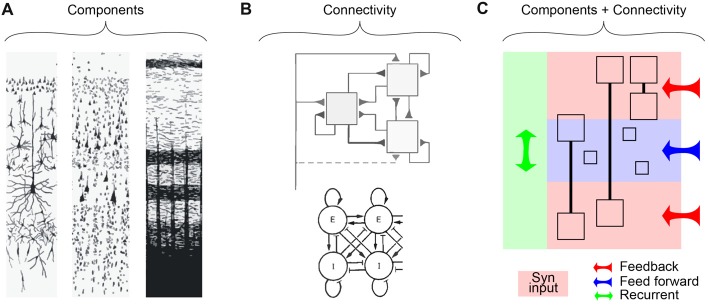
Shifting perspectives about cortical layering. **(A)** Nineteenth century descriptions of the cortex emphasizing the components in terms of cytoarchitecture and axonal projections. Left, Golgi stain; Middle, Nissl stain; Right, Weigert stain (Heimer, 1994, adapted with permission). **(B)** Functional views emphasizing connectivity in the cortex. Top, “canonical circuit” of the neocortex (Douglas et al., 1989, adapted with permission); Bottom, balance of excitation and inhibition (Wilson and Cowan, 1972, 1973; adapted with permission). **(C)** A simplified representation of the class of description needed to describe emerging data in terms of the underlying architecture of a layered structure such as the cortex. Here, components (i.e., neurons) are described in terms of their principle dendritic compartments (boxes) and the way they span the cortical layers. Small boxes represent neurons that putatively can be still described as point neurons (although this is yet to be established). The proposed combined perspective should take into account the broad influences of long- (Feedback, red and Feed forward, blue) and short-range (Recurrent, green) connectivity and the functional components (compartmental neurons) that span multiple layers simultaneously.

Neuroscience has seen a dramatic evolution since the early anatomical investigations of the 19th and 20th centuries. With the discovery of different ways to stain brain tissue, the original emphasis was on cataloging the components of the brain (Figure 1A, “Components”). This approach was enormously successful in describing the structure of the cerebral cortex as a laminar structure based on the cytoarchitecture. Reaching its zenith in the first half of the 20th century (Defelipe et al., 1988), ever more detailed descriptions of the precise configuration of cells and axons promised to explain the function of the cerebral cortex. However, with the development of techniques for recording activity directly from the neurons of the brain, the original focus on a faithful anatomical description gave way to more simplified descriptions of the functional anatomy, i.e., organization and connectivity of structures (Hubel and Wiesel, 1962; Mountcastle, 1978). Here, various models were offered for describing the organization of the cortex (Figure 1B, “Connectivity”). The increased emphasis on connectivity often came at the expense of the complexity of the components, mostly treated as simple point neurons. In this article, we take the view that a full description of a laminar structure like the cortex will require the successful marriage of both the components and the connectivity that captures an adequate description of both aspects and their interplay.

## What Defines a Layer as a Subunit of Function?

Treating neurons as single points comes at a great price in a layered structure like the cortex. In fact, neurons have complicated morphologies and properties that typically span multiple layers. It is conventional to refer to “cells of a specific layer.” The description “layer 5 neuron,” for instance, conventionally refers to a neuron whose *cell body* lies in layer 5. However, the properties of this cell are distributed across multiple cortical layers throughout their dendrites and axon. The merging of components and connectivity in a description of the layered cortex needs to take into account the location and influence of synaptic inputs and the resultant electrical events that occur within layer-spanning neurons (Figure 1). For example, layer 5a and layer 5b pyramidal cells differ predominantly in their properties defined by the apical dendrites (Major et al., 2013).

A description of the cortex emphasizing connectivity between simple components—named after the cell body location—is seductive, particularly to computational neuroscientists, but also to physiologists who measure the “activity” of a neuron by recording from the cell body. It turns out that the neurons generate action potentials very close to the cell body, which is also the location that is most accessible for recording the neuron output. However, there is no functional consequence to this fact from the perspective of an input-output description of the cortical circuit and in particular for ascribing functionality to layers (see Box 1). Neither the input nor the output is actually best described as located at the cell body. Both the inputs (postsynaptic potentials) and the outputs (transmitter release) of a single neuron could literally occur in any and all of the layers of the cortex. From this perspective, there is no such thing as a “L5 pyramidal neuron.” Nevertheless, there is a general correspondence between the cytoarchitecture, and between the layering apparent due to cell bodies and the layering of axonal terminations (Figure 1A, middle and right).

Box 1 Re-evaluating the somato-centric perspective in layered structures. Since Cajal (1894) proposed the Neuron Doctrine, it has been clear that information (by and large) flows uni-directionally across neurons in the nervous system. This has lead to a pervasive and reductive description of how neurons operate that is apparent both in the way most neuroscientists talk about “activity” in the brain and the way the operation of neurons is formalized **(A)**. Here, axon terminals are frequently described as the “input” to a neuron, which is usually represented only as a “conceptual cell body” that emits “output.” This perspective has survived more than a century since Cajal because, we argue, it fits the intuition that cell bodies are both physically prominent and provide a convenient locus for recording action potentials. In fact, the physical situation, which is well understood but frequently overlooked, is that the output of a neuron almost always manifests as the release of transmitter *at the axon terminals* whereas the input is best described as synaptic currents located directly abutting the terminals **(B)**. From a methodological point of view, this means that methods such as fMRI are typically describing energy related to release of transmitter at axon terminals (see main text) and local field potentials describe synaptic currents located nearby. With the advent of methodologies that can more precisely resolve the layering of the cortex (e.g., high-res fMRI), it becomes necessary to shift perspectives in order to interpret the signals. Because of the close apposition of input and output, the choice of label for synapses reduces quickly to semantics and it is not the argument here that the nomenclature needs to change. However, the current “somato-centric” perspective that we owe originally to Cajal cannot be used to explain the data emerging with modern techniques. It is increasingly well understood that the “function” of a neuron (i.e., the transformation from input to output) occurs via the process of dendritic integration that in the cortex frequently occurs in active and layer-spanning dendritic trees **(C)**. Thus, the computation of a cortical neuron is actually a complex spatio-temporal phenomenon that transforms inputs arriving over various layers to output delivered to various other layers **(D)**. In this transformation process, the cell body specifies neither the location of the processing nor the output of the neuron and could in principle be collapsed to a dimensionless node in any specific layer without substantially changing the input/output function of the neuron. It is therefore not correct from a functional perspective to attribute the “activity” of a cell to the layer in which the cell body is located. The interpretation of BOLD signals from high-res fMRI recordings, for instance, cannot be attributed simply to the spiking neuronal activity occurring in the same layers as the BOLD signal, but rather to the summed mostly post synaptic membrane potential fluctuations of dendrites whose cell body is elsewhere.

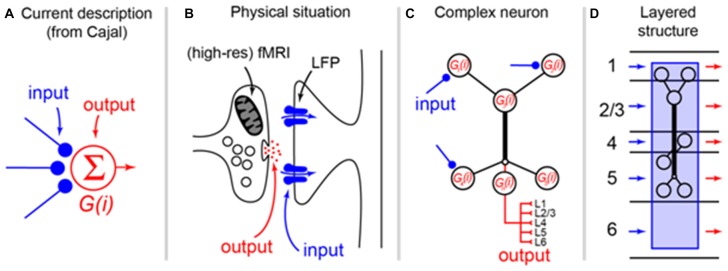

The last few decades have witnessed a huge increase in our knowledge about the properties of dendrites and how they integrate synaptic input (Spruston, 2008; Major et al., 2013; Grienberger et al., 2015). For instance, we now know that pyramidal neurons in the cortex have various types of local dendritic spikes (Na^+^, Ca^2+^ and NMDA) and diverse distributions of ion channels that influence the propagation and local integration of synaptic potentials. It is now clear that these neurons are more complex than simple point neurons but a canonical description has yet to emerge. Ideally, we should be able to describe neurons in functional terms with reference to the number of compartments, the organization of input and output and their relationship to the layering of the cortex. Nevertheless, with the advent of optical methods it has become possible to directly image activity in particular axons, in dendrites and in cell bodies situated in particular layers (Svoboda et al., 1997; Petreanu et al., 2007; Jia et al., 2010; Andermann et al., 2013; Kim and Kastner, 2013) which promises to increase our understanding. Functional MRI, measuring BOLD contrast which is a combination of blood flow, blood volume and blood oxygenation, is better linked to neuronal activity by summed energy consumption than by spiking neuron output. With the increased spatial resolution of fMRI in recent years, BOLD is now measured at different cortical depths and can therefore be used to characterize the summed energy consumption in different layers of cortex. Great advances have also been made in anatomical approaches for examining brain connectivity at all scales (Bassett and Sporns, 2017). Notably missing so far is a coherent integration of these revolutionary thrusts in the neurosciences.

In summary, our perspective on cortical layering is the following:

1. The biophysical/computational input/output properties of the components of the cortex are complex and are spatiotemporal in nature often spanning several layers.
2. The cortical layer in which the cell body of a neuron is located has little or no ramifications for computing the input/output function of that neuron.
3. Understanding *any signal recorded from the cortex* needs to take these facts into account, preferably with some model or theory that accounts for the underlying structure/function.

## Combining the Components and Connectivity in a Description of the Cortex

To date, models of cortex that include the laminar structure are only a small proportion of the total and these models tend to ignore the dendrites or treat them only cursorily (Spratling and Johnson, 2001; Spratling, 2002; Raizada and Grossberg, 2003; Thomson and Bannister, 2003; Grossberg, 2007; George and Hawkins, 2009). By using simplified components, such models may fail to successfully account for the interactions across layers that are sometimes carried out within the neurons themselves. To achieve this, a description of the full repertoire of dendritic properties with respect to the different cortical lamina will be necessary. The final goal of such a model is a description of how the inputs between and within cortical areas are transformed into laminar-specific output throughout the system.

**Figure 2:**
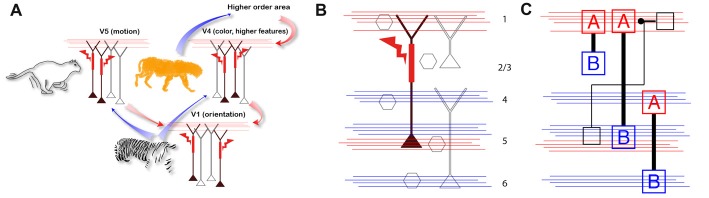
Approaches for combining components with cortical layering. **(A)** A hypothesis for the possible ramifications of the associative properties of cortical pyramidal neurons with dendritic calcium spikes at the network level (adapted with permission from Larkum, 2013). Here, the active properties of the apical dendrites associate feed-forward and feedback information streams arriving at different layers. Here, blue arrows indicate feed forward information streams and red arrows indicate feedback. **(B)** Missing components (gray) needed for an expanded theory of the one shown in part A which should include the intrinsic properties of neurons, dendrites and synaptic inputs. Feedback and feed forward axonal input indicated with red and blue lines, respectively. **(C)** Example of abstractions of neurons needed for new theories within the new perspective. Here, A = dendrites, B = Somata.

A simple example of such a model was proposed in a recent hypothesis of how the dendritic calcium spike in pyramidal neurons might associate feed-forward and feedback information streams arriving at different cortical layers (Figure 2A; Larkum, 2013). Here, the pyramidal neuron acts like a coincidence detector for simultaneous input to the upper and lower cortical layers with Ca^2+^ spikes facilitated by back-propagating action potentials (BAC firing; Larkum et al., 1999). Whether or not the cortex operates exactly in this fashion is still an open question. Evidence in favor of this particular hypothesis was recently demonstrated by showing that the threshold for perception correlates with dendritic calcium spikes in layer 5 pyramidal neurons and that down-regulating the calcium spikes suppressed perception at threshold stimulus levels (Takahashi et al., 2016). On the other hand, alternative explanations have been offered to explain exactly which inputs lead to calcium spike firing. For instance, feedback inputs also arrive in the lower layers and possibly triggering the BAC firing mechanism on their own (Manita et al., 2015, 2017). However, the fact remains that the calcium spike apparently has an effect on the perceptual threshold that cannot be explained by models with point neurons. The main upshot is that it is necessary to have an account of how cellular processes such as local spikes and subcellular propagation (Major et al., 2013; Stuart and Spruston, 2015) interact with inputs and under what behavioral circumstances in order to interpret any given recordings and explain complex behavior.

In conclusion, we argue that it is fundamentally important first to examine all cell types of the cortex and describe and encapsulate their properties and the way they integrate synaptic inputs (Figures 2B,C). This task can be started *in vitro* but eventually must be validated *in vivo* under awake behaving conditions. Second, having obtained the biophysical facts about the components of the cortex, it will be necessary to develop abstractions of these components so that their functionality can be captured in a model. Third, this information has to be combined with the connectivity of the cortex so that the influence of particular inputs can be included in the model. With this information in hand, it becomes possible to interpret laminar-specific data collected from the cortex whether it comes in the form of electrical recordings from particular cells in particular layers or imaging of brain activity at various levels of resolution and various cortical depths.

## A Case Study—Ultra High-Resolution fMRI

**Figure 3:**
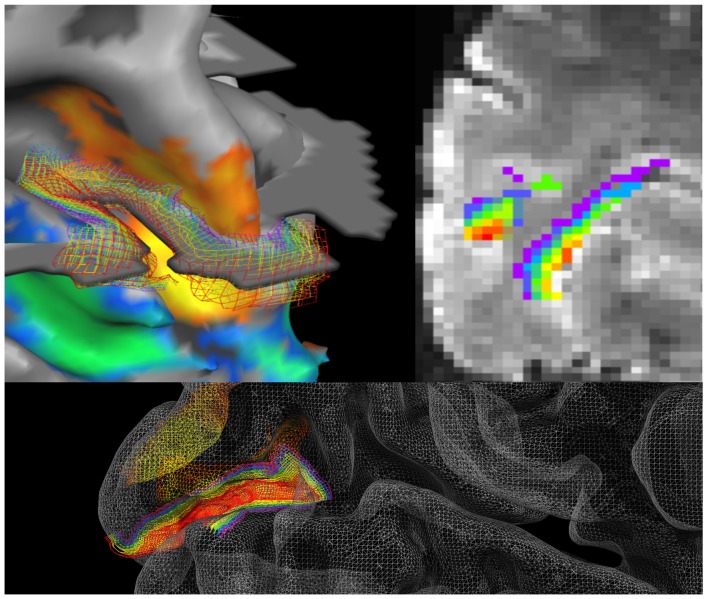
Cortical surface reconstruction. Left hemisphere in the human acquired with high resolution 7T fMRI, overlaid with grid lines depicting cortical depths from superficial (red) to deep (purple) layers; (left upper) at the boundary of V2 and V3 right and lower indicate cortical depth levels in V1 (Muckli et al., 2015, adapted with permission). With this method, voxels in fMRI are labeled depending on their cortical depth and incorporated into depth-specific analyses. In the above example, the fMRI data is at 0.8 mm^3^. Even though the bands of cortical depth level measured with fMRI are still insufficient to separate all six anatomical layers of cortex, there are important gradients that are functionally different in their processing of internal mental states, which we can capture by separating the layers into upper, middle and lower (i.e., by separating feedforward and feedback processing).

To elucidate this argument, we take a case study using the relatively new technique of ultra high-resolution fMRI that allows researchers to investigate brain activity non-invasively in human subjects with the ability to separate different depth layers in cortex (Figure 3). With this technique, blood oxygenation level-dependent signals (BOLD) can be measured for the top, middle and bottom thirds of the cortex. This, in turn, approximates to the layers known to roughly segregate according to synaptic inputs from feedback vs. feed forward connectivity (Petro and Muckli, 2016, 2017). We take this example because it highlights both the gains that can be made by demanding informed interpretations and the pitfalls of proceeding without them. It is ideal as a case study because, unlike traditional fMRI, this is a technique that can be applied to, and in our opinion calls for, an understanding of the operation of the cortex from a laminar perspective (Muckli et al., 2015; Huber et al., 2017; Lawrence et al., 2017).

But what conclusions about high-res fMRI recordings are valid? For example, suppose higher BOLD signals are detected at one depth vs. another? Signals in fMRI studies are often conflated with neuronal “activity.” This is problematic on a number of levels. BOLD signals largely reflect the energy consumption of neural processing that require oxygen and glucose rather than a direct reflection of neuronal activity. Therefore, the BOLD signal most likely reflects locations of high synaptic activity (Logothetis et al., 2001; Viswanathan and Freeman, 2007; Logothetis, 2008) because the process of synaptic transmission has the largest energy requirement. Importantly, the activity of excitatory and inhibitory neurons would simply combine on this view despite their opposite affects. Nevertheless, even with this understanding, it is often implied that high synaptic activity translates to high post-synaptic activity. However, the post-synaptic effect will depend on the complexities of the post-synaptic targets. For instance, large synaptic input to the upper layers that might occur due to an increase in feedback information would have a complex relationship to the firing of neurons that have distal dendrites projecting to the upper layers and their cell bodies and proximal dendrites in deeper layers. The major effect of such activity might be to depolarize the apical dendrites of pyramidal neurons whose cell bodies are in lower layers and whose axons terminate mostly in different cortical areas. Or, the same input may activate dendrites targeting inhibitory neurons that have the opposite effect on the dendrites of pyramidal neurons. Furthermore, the relative proportion and timing of inputs to different cortical layers will lead to further complexities that are mostly determined by the biophysics of the cells themselves. Some of these predictions can be guessed already from our knowledge of the operation of components such as layer 5 pyramidal neurons but even for these well-studied neurons their description is probably still not complete. There is not yet a consensus description of these neurons that encapsulates their function under many simple input patterns. For other neurons of the cortex the situation is even less clear such that predictions of what happens under various different conditions remain wild guesses at best. Furthermore, most of the biophysical information we have so far about the operation of subcellular membrane potential dynamics is derived from recordings from rodent neurons under controlled conditions *in vitro*. What will happen in human neurons that are much larger (Mohan et al., 2015; Deitcher et al., 2017) and may have different properties (Eyal et al., 2016)? Good predictions about what signals should be expected using ultra high-resolution fMRI in humans during cognitive tasks will require a coherent theory of what occurs with different patterns of laminar input in different areas under different conditions.

Such a theory would be timely because layer-dependent measures using ultra high-field brain imaging are a rapidly developing field in human cognitive neuroscience (Lawrence et al., 2017; De Martino et al., 2018). At present, these high-resolution brain imaging studies have not tested the theories and tasks of their standard brain imaging counterparts at 3 Tesla. We anticipate studies of learning, memory, multisensory processing and consciousness which are surely forthcoming at laminar resolution. These studies would build on a number of successful proof-of-concept findings, measuring, for example, BOLD activation in layers of primary visual cortex in response to its preferred stimulus of contrast-reversing checkerboards (De Martino et al., 2013); cerebral blood volume and BOLD signal changes in layers of human primary motor cortex during finger tapping (Guidi et al., 2016); and layer-dependent population receptive field sizes in human primary visual cortex (Fracasso et al., 2016). While these studies provide conceptual advances in human sensory and motor systems, they do not address the laminar influence of feedback vs. feedforward sources, or in functional terms how cognitive processing interacts with sensory processing. For example, when viewing natural scene images, cortical feedback transmits the brain’s internal model of the scene back to primary visual cortex, predominantly the superficial layers (Muckli et al., 2015). In superficial layers, this feedback is detected with fMRI as changes in the activation distribution measured in multi-voxel pattern activity. Another finding relevant for the question of top-down feedback modulation of vision to cortical layers, is the demonstration that the deep layers of V1 are more active for perceptual filling-in of contours, a form of modal completion, (Kok et al., 2016). This was shown as a univariate increase of activity in deep layers. Taken together, both studies suggest how cognitive function interacts with sensory processing on a sub-neuronal level; top-down expectations are present in the dendritic tree of superficial layers of V1, and when combined with specific contextual information they can trigger activity in deeper layers and the illusory percept of visual contour filling-in. Although this interpretation of both studies has not yet been tested directly, it highlights the potential of ultra-high-field fMRI in detecting the functional properties of sub-neuronal compartments. Combined multi-method experiments are ongoing to establish this interpretation across species and scales. Another example of top-down modulation in layers of sensory cortex is provided by De Martino et al. (2015) who show that attention sharpens the representation of acoustic information mainly in the superficial layers of human primary auditory cortex. All of these studies give rise to the question of the circuit and systems level mechanisms of top-down, feedback processing. In a first demonstration of its kind, human laminar fMRI was used to derive information flow between cortical areas (Huber et al., 2017). Using BOLD and CBV (cerebral blood volume) measures, the authors revealed somatosensory and premotor input in upper layers of M1 and cortico-spinal motor output in the deeper layers. Moreover they found layer-specific functional connectivity of M1 and somatosensory and premotor areas using laminar resting-state fMRI. Technical advances in high-resolution fMRI will ensure that we can improve spatial coverage, meaning in future we can image even larger areas of cortex, that are capturing information flow between additional communicating regions.

Underpinning these functional cognitive brain imaging studies, there exists a broad field advancing laminar differences in cerebral blood flow, cerebral blood volume, neurovascular coupling, vascularity, positive and negative BOLD, blood flow regulation, and comparison to electrophysiology, not to mention laminar-specific data acquisition and analysis strategies (Goense et al., 2016; Self et al., 2017; Kashyap et al., 2018; Kemper et al., 2018). All of these topics will be bolstered by (and are essential components of) what we propose here: the necessity of interpreting layer-specific fMRI data with knowledge of the laminar distribution of inputs and layer-spanning cellular compartments. We need a theory that incorporates the complex properties of neuronal components whose dendrites and axons span multiple layers because, while these current studies already highlight the tremendous potential of this technique for the study of cognitive function in higher brain areas, it will remain difficult to fully disentangle the effects of feed forward and feedback inputs in human cortex without understanding the underlying biophysics. The interpretation of the layer specific fMRI signal therefore requires an *a priori* theory of cortical function, describing the functional consequences of the laminar distribution of synaptic inputs for a neuron. Even for studies in rodents where there is a constant evolution in the methods, and where the temporal and spatial resolution is much higher than with fMRI, the ability to collect large data sets is ultimately meaningless without a theory (Jonas and Kording, 2017).

The promise of high-resolution fMRI is in allowing a window into the neuro-computational unit of cortical layers during the most elaborate cognitive states, for example, emotion, inner speech, empathy and mental time travel. Non-invasive approaches are crucial because we are not close to being able to image activity at a cellular or subcellular resolution in human beings, or to applying experiments where one manipulates specific pathways using approaches like optogenetics. On the other hand, this situation does not mean that the underlying cellular and subcellular dynamics can therefore be ignored without influencing the interpretation. If brain imaging can reveal a functionally-relevant meso-scale abstraction of the synaptic inputs in micro-scale neuroscience, this will amount to an important breakthrough. At some point in the very near future it may then become possible to talk about consequences of activity in cortical areas during cognitive tasks in the context of feed forward and feedback inputs, and activity in the different layers.

## Linking Complex Components With Laminar Connectivity—What Still Needs to be Done?

In our opinion it would be unwise to use oversimplified assumptions (such as point neurons) as a substitute for our ignorance. Fundamental questions remain regarding many aspects of cortical layering and how this interacts with the complexities of layer-spanning neurons with active dendrites. For instance, we lack a satisfactory account of what role “basal” dendrites play, that are largely limited to the same or adjacent layer as the cell body. It has been suggested that local dendritic NMDA spikes in these dendrites offer multiple, independent integrations of incoming signals (Mel, 1992; Polsky et al., 2009). In fact, this observation has been extended to the tuft dendrites of pyramidal neurons (Larkum et al., 2009; Palmer et al., 2014) and may be a generalization for thin dendrites of other neurons (Lavzin et al., 2012). In general, however, we still lack the specific and necessary information to accurately describe the input/output function of most neurons and their relationship to the layering of the cortex. In fact, most modern methods do not measure either input or output directly (see Box 1) but rather action potential activity at the cell body. In cases where interesting processes occur in the dendrite but the cell fires no action potential, the underlying events might still be detectable with methods such as high-resolution fMRI.

Perhaps most importantly, the learning rules for synaptic connectivity are yet to be linked conclusively to the full range of intrinsic activity and laminar circuitry. There is good evidence that dendritic Na^+^ and Ca^2+^ spikes influence synaptic plasticity (Kampa et al., 2006; Sjöström and Häusser, 2006; Losonczy et al., 2008) as well as NMDA spikes (Gordon et al., 2006; Brandalise et al., 2016). Nevertheless, an integrated theory of how all these isolated phenomena combine to result in network rewiring is still lacking. Recent intriguing results from the Magee group suggest that some combination of these intrinsic dendritic properties may be transformative in explaining learning in the hippocampus (Bittner et al., 2015; Grienberger et al., 2017). In the neocortex there is accumulating evidence that plasticity occurs at feedback and 2nd order thalamic synapses on to the apical tuft dendrites of L5 neurons in the upper layers of the cortex (Gambino et al., 2014; Cichon and Gan, 2015; Miyamoto et al., 2016). All these forms of plasticity depend on this activation of intrinsic dendritic activity. The exact conditions or rules that control this kind of learning are still being determined but it is clear that they cannot be understood or described without reference to both the laminar pattern of connectivity and the intrinsic properties of the neurons that process these inputs in distinct compartments lying in distinct cortical lamina.

At the present time, most laboratories still focus on recordings from cell bodies and some even still report data without reference to the cortical layer or cell type from which they are taken. Two-photon imaging approaches have opened up the possibility of collecting data from compartments other than the cell body but recording data from anything other than cell bodies is still the exception rather than the rule. In the meantime, improvements to 2-photon imaging (Ji et al., 2016; Papadopoulos et al., 2016), other methodologies such as prisms inserted into the cortex (Andermann et al., 2013) and high-resolution fMRI now make it possible to include laminar-specific information. Standard methodologies such as vertically oriented linear arrays like Michigan Probes have long been useful for probing laminar issues (BeMent et al., 1986; Cauller and Kulics, 1991) and can be used to probe the relative influence of feed forward and feedback influences in the cortex (van Kerkoerle et al., 2017). It was recently shown that intrinsic excitability of pyramidal neurons such as calcium spikes are also easily detected by these devices (Suzuki and Larkum, 2017).

In summary, it is now possible to move beyond a simple description of cortex in terms of point neurons and point-to-point connections in favor of a richer understanding that includes vertical features such as axonal termination layers and subcellular compartments spanning several layers. We have learned enough about the properties of particular neurons such as the layer 5 neuron to be able to say for certain that their active dendritic properties interact with the location of synaptic inputs in a very complex but important way. In our view, it is essential to investigate these features in all neurons and synaptic input pathways in order to understand the layering of the cortex.

## Author Contributions

ML, LP, RS and LM wrote the article.

## Conflict of Interest Statement

The authors declare that the research was conducted in the absence of any commercial or financial relationships that could be construed as a potential conflict of interest.
